# Sugar-Sweetened Beverage Tax Intensification, Beverage Affordability, and Food-Security Safeguards: Global Evidence from 2022–2024

**DOI:** 10.3390/foods15183343

**Published:** 2026-09-20

**Authors:** İbrahim Olgun, Musa Gün

**Affiliations:** Faculty of Economics and Administrative Sciences, Recep Tayyip Erdoğan University, Rize 53100, Türkiye; musa.gun@erdogan.edu.tr

**Keywords:** sugar-sweetened beverage tax, excise taxation, food security, healthy-diet affordability, tax incidence, fiscal policy, entropy balancing, open data

## Abstract

Sugar-sweetened beverage (SSB) taxes aim to discourage unhealthy consumption, but their implications for broader food affordability also need monitoring. We examine whether changes in SSB excise-tax shares between 2022 and 2024 were associated with beverage affordability, healthy-diet affordability, and food insecurity. The analysis links WHO beverage-tax data with FAO food-system indicators and World Bank controls. First-difference models include 83–113 countries, depending on the outcome, with adjustment for multiple testing. A one-percentage-point increase in excise share was associated with a 0.0023-log-point change in SSB income burden (95% confidence interval −0.0349 to 0.0395). This estimate is small and imprecise. The PPP-price association depends on an anomalous source record, while the three food-system outcomes show no statistically precise associations after multiplicity adjustment. Because food insecurity is measured over overlapping three-year windows, it serves as a contextual indicator. The findings establish neither that tax intensification is ineffective nor that adverse food-system effects are absent. They support joint monitoring of beverage affordability and food access and identify a need for longer panels and household-level evidence.

## 1. Introduction

Governments increasingly use taxes on sugar-sweetened beverages (SSBs) to discourage unhealthy consumption. Their adoption has expanded globally, although tax rates, product coverage, and protection against inflation still differ markedly across countries [1,2,3,4,5]. Evidence links SSB consumption to weight gain and cardiometabolic disease [6,7,8,9]. Taxing these drinks can reduce purchases, but the consequences for households depend on their initial consumption, subsequent choices, and the use of public revenue [10,11,12,13,14,15,16,17,18,19]. An assessment of affordability must therefore consider access to a healthy diet as well as the price of the taxed beverage.

National evaluations show that SSB taxes operate through several mechanisms. In Mexico, purchases of taxed beverages fell by an average of 6% in the first year, with larger declines among households of lower socioeconomic status [20]. Later evidence found that the response persisted [21]. The United Kingdom’s tiered Soft Drinks Industry Levy prompted reformulation: substantially fewer products remained above the lower sugar threshold, and producers passed only part of the levy into the prices of high-sugar drinks [22]. Studies of South Africa’s sugar-based levy separately examine beverage prices and purchasing responses [23,24]. Evaluations from Chile, Saudi Arabia, Barbados, and US jurisdictions cover further differences in rates, taxable products, and retail conditions [25,26,27,28,29,30,31,32,33]. Across this evidence, reviews report lower purchases on average but considerable variation in pass-through and substitution [10,11,12,13,14,15,34,35,36]. Such variation is plausible given differences in tax design, reformulation before implementation, cross-border shopping, and access to untaxed substitutes. A price response observed in one market consequently offers a limited guide to what another country should expect.

Beverage affordability and healthy-diet affordability describe different constraints. The beverage indicator relates the cost of a fixed volume of a benchmark drink to income; the healthy-diet indicator concerns the cost of a nutritionally adequate basket and the resources needed to obtain it [37,38,39,40,41,42,43,44,45,46,47,48,49]. Raising the relative price of SSBs need not make healthy staples more expensive. Equally, food inflation can restrict access to a healthy diet when beverage taxes remain unchanged. SSBs offer a useful policy case because they are widely taxed and internationally comparable monitoring data are available. Their share of expenditure varies across households and countries, so they cannot represent the entire food budget.

We examine whether larger increases in SSB excise-tax shares from 2022 to 2024 accompanied changes in beverage affordability and broader food-access indicators. We link the two WHO tax and price waves to FAO measures of healthy-diet cost, healthy-diet unaffordability, food insecurity, and food prices, together with World Bank controls [2,3,4,50,51,52]. A separate cross-sectional analysis assesses how the 2024 excise share and documented tax-design features relate to the benchmark beverage’s income burden in that year.

The study brings the intended change in beverage affordability and three potential food-system safeguards into a common monitoring framework. Corrective-tax and incidence arguments guide the selection of outcomes [53,54,55,56,57]. Robust inference, adjustment for multiple testing, and balancing on observed covariates help assess how much the country-level evidence can establish [58,59,60,61,62,63]. The resulting estimates describe adjusted associations. They do not identify causal tax effects or test whether food-security outcomes are equivalent across policy settings.

## 2. Analytical Framework and Objectives

### 2.1. Corrective Taxation, Tax Incidence, and Affordability

The analytical framework connects the research question to the five outcomes and the supplementary tax-design analysis. Section 3 sets out their measurement and estimation. Corrective taxation addresses external costs, consumer decision-making, and distributional concerns [17,53,54,55,56]. Whether a statutory tax reaches retail prices depends on market structure, demand, and firms’ pricing decisions [55,57]. The three objectives below organize our descriptive analysis. They are not preregistered causal hypotheses, and an imprecise estimate cannot establish that a food-security safeguard has been met.

The WHO measures beverage affordability by relating price to average income at the country level. Let P_it_ denote the local-currency price of 50 L of the benchmark SSB in country i and year t, and let Y_it_ denote nominal GDP per capita. Income burden is defined as:

(1) $$B_{it}=100\times \frac{P_{it}}{Y_{it}}$$

Equation (1) expresses the cost of 50 L of the benchmark beverage as a percentage of nominal GDP per capita. Higher values denote lower affordability. Holding the purchase volume constant allows comparisons between beverage-price movements and average income growth [37,38,39]. This benchmark does not record what households actually buy, spend, or pay in tax, and it cannot establish the burden borne by low-income consumers. Its denominator is aggregate GDP per capita, which differs from household disposable income. When nominal income and beverage prices rise at similar rates, the indicator may show little change.

Analytical objective 1. Examine the association of excise-share changes with changes in the benchmark beverage’s PPP price and income burden. A statutory levy may raise retail prices. However, the exposure used here is a tax-to-price ratio, and the income-burden measure also depends on aggregate income. These features prevent an unambiguous prediction about the direction of the estimated associations.

### 2.2. The Food-Security Safeguard

The claim that consumption taxes are regressive usually concerns the proportion of current income paid by lower-income consumers. This accounting measure matters, but it excludes behavioral responses, health gains, and the use of tax receipts [13,17,19]. It may also obscure the distinction between a targeted change in relative prices and general food inflation. For the present analysis, the safeguard question is whether SSB taxation coincides with reduced household access to a healthy diet.

FAO’s CoAHD framework estimates the least-cost basket consistent with food-based dietary guidelines. Work on retail prices in 177 countries, the EAT–Lancet diet, poverty thresholds, and a standardized Healthy Diet Basket has refined its use for international monitoring [43,44,45,46,47,48,49]. CoAHD provides a comparable cost floor rather than observations of household expenditure. We combine it with the prevalence of healthy-diet unaffordability and the Food Insecurity Experience Scale to distinguish the basket’s price, the resources needed to buy it, and experienced difficulties in obtaining food.

An excise confined to SSBs does not directly tax the healthy-diet basket. Indirect effects may arise if households substitute other foods, retailers change prices, or governments direct tax receipts to water access, school meals, or food support [13,16,19,41,42]. These mechanisms can work in different directions. Since the data do not measure household substitution or benefits financed by revenue, we use the broader outcomes for monitoring rather than to test the full transmission process.

Analytical objective 2. Examine whether changes in excise shares accompany changes in healthy-diet cost, healthy-diet unaffordability, and moderate or severe food insecurity. The three measures cover the price of the healthy basket, the resources needed to obtain it, and reported difficulties in accessing food. An imprecise association leaves open the possibility of either improvement or deterioration.

### 2.3. Why Design Can Matter More than Tax Presence

The way an excise is structured may shape responses as much as its presence [1,5,16]. Specific taxes apply to volume or sugar content, whereas ad valorem taxes apply to value. The UK evidence shows how sugar-content tiers can encourage manufacturers to reformulate drinks [22]. Indexing a specific levy to inflation preserves its real value; maintaining its strength relative to income also requires adjustment for income growth [2,37,38]. Exempting unsweetened water leaves a healthier substitute untaxed, provided that households can obtain safe water in practice [2,3]. How governments allocate the revenue can influence net benefits and public support. Earmarking alone, however, does not determine how retailers pass the tax into prices [3,17,19,57].

We summarize documented tax design using six binary features: an SSB excise, sugar-based tiers, automatic adjustment, an exemption for unsweetened water, a specific excise component, and earmarked revenue. Each feature contributes one point to the checklist. Equal weighting makes the coding straightforward to inspect, but it does not assign an empirically estimated benefit to any feature. Nor does the checklist capture enforcement, the amount of revenue recycled, or whether adjustment keeps pace with both inflation and income growth. The replication dictionary provides the coding definitions.

Analytical objective 3. Describe the associations of the 2024 excise share and design checklist with log SSB income burden in 2024. This supplementary analysis examines tax architecture at one point in time; it does not test the two-wave relationship. Models for individual components and for excise countries alone assess whether the aggregate checklist results depend on its composition or the sample.

## 3. Materials and Methods

### 3.1. Open Data and Country Linkage

Table 1 summarizes the data sources and their analytical roles. The WHO workbook provides a country-wave record of the price and tax components of an internationally comparable SSB, together with design fields for non-alcoholic beverage categories. The country frame includes 195 countries. Excise-share observations are available for 135 countries in 2022 and 160 in 2024, with 124 observed in both waves. The corresponding income-burden indicator is available for 129 countries in 2022 and 155 in 2024.

The FAOSTAT releases identified in the replication workbook provide healthy-diet costs and unaffordability for 2022 and 2024 [43,44,45,46,47,48,49,50,51]. For moderate or severe food insecurity, we compare the published prevalence estimates for 2020–2022 and 2022–2024. These windows overlap in 2022 and include observations outside a distinct post-policy period. Their difference is therefore a contrast between smoothed monitoring estimates, not an annual change or an independent before-and-after comparison. Income shocks, conflict, weather, and transfers may also contribute to the observed contrast. We calculate annual food consumer price indices by averaging the available monthly values in each year. World Bank data provide 2021 PPP GDP per capita, the urban population share, and regional classifications [52].

We normalize country names and match them to World Bank metadata. Manual aliases address naming differences only, including “Türkiye,” “Côte d’Ivoire,” and “Lao People’s Democratic Republic.” The crosswalk retains both source and matched names for inspection. We do not impute missing outcomes, tax shares, or prices.

### 3.2. Variables

Our main exposure is the percentage-point change in excise tax as a share of the benchmark beverage’s retail price. Because price enters the denominator, we treat this measure as an exposure rather than an exogenous treatment:

(2) $${\Delta ExciseShare}_{i}={ExciseShare}_{i,2024}-{ExciseShare}_{i,2022}$$

Equation (2) describes the within-country change between the two WHO waves. We also use the change in total-tax share as an alternative exposure. The supplementary group comparison defines an “intensifier” as a country with an excise-share increase of at least two percentage points. “Stable” countries have absolute changes below two points. This analyst-chosen cutoff separates larger movements from near-stability; it is neither a theoretical discontinuity nor a validated boundary for measurement error. Crossing it does not establish that a statutory reform occurred. We repeat the comparison at cutoffs of 1, 3, and 5 points, retaining the symmetric definition of stability. The main continuous-exposure models require no cutoff. Since excise share can move through the tax amount, retail price, or both, its association with PPP price cannot identify pass-through.

We use five outcomes to examine beverage affordability and broader food access. Changes in log PPP SSB price describe the targeted product’s price, while changes in log SSB income burden also account for average income. Log healthy-diet cost tracks the least-cost healthy basket. The change in healthy-diet unaffordability, measured in percentage points, describes how the share of people without sufficient resources for that basket has shifted. Moderate or severe food insecurity captures reported difficulties in obtaining food and is also expressed as a percentage-point contrast. This last outcome prevents a stable beverage indicator from being treated as evidence of adequate food access, although its overlapping observation windows align less closely with tax changes. Each outcome thus addresses a different aspect of the monitoring question; none provides a complete measure of household welfare. Log differences approximate proportional changes, whereas prevalence differences retain percentage-point units.

The covariates are log 2021 PPP GDP per capita, the 2021 urban population share divided by ten, the 2022–2024 log change in the food consumer price index, and World Bank region indicators. Baseline income and urbanization account for structural differences across countries. Contemporaneous food inflation adjusts for broader price movements but could itself respond to a wider fiscal package. We therefore estimate specifications with and without this control, without assuming that it necessarily precedes the exposure.

### 3.3. First-Difference Model and Inference

For country i, the primary model is:

(3) $${\Delta y}_{i}=\alpha +\beta {\Delta T}_{i}+\gamma^{\prime }Z_{i}+\lambda_{r}+\varepsilon_{i}$$

In Equation (3), Δy_i_ denotes an outcome change and ΔT_i_ the change in excise share. Z_i_ includes baseline log income, urbanization, and the log change in the food consumer price index; λ_r_ denotes the region indicators. First differencing removes country-specific levels that do not change over time. It cannot eliminate unobserved policy or market shocks that do change. We therefore interpret β as an adjusted association rather than a causal average treatment effect.

We estimate the models by ordinary least squares, using HC3 standard errors and two-sided Student t reference distributions with residual degrees of freedom [58,59]. Primary resampling inference uses 9999 Rademacher wild-bootstrap draws under the null and HC3 studentization [61]. We apply the Benjamini–Hochberg adjustment separately to the five HC3 *p*-values and to the five wild-bootstrap *p*-values [62]. The approximate minimum detectable coefficient at 80% power equals 2.80 times the HC3 standard error, based on a normal approximation for a two-sided 5% test. This quantity describes sensitivity to a one-point change in exposure; it is not an equivalence margin. The archived Freedman–Lane permutation results [63] remain secondary diagnostics, since unequal leverage and heteroskedasticity cast doubt on residual exchangeability.

### 3.4. Robustness and Balanced Comparison

We assess sensitivity by omitting the food-CPI control, excluding Sierra Leone, excluding excise-share cuts of at least two points, substituting total-tax-share change for excise-share change, and winsorizing the exposure and outcome at the 2.5th and 97.5th percentiles. For each outcome, the winsorization thresholds come from its baseline complete-case sample; controls remain unchanged. We also estimate the income-burden model after leaving out each country in turn. Appendix A (Table A1 and Table A2) contains the full results. A direct check of the WHO workbook (on 15 September 2026) found Sierra Leone’s 2022 PPP price of 0.0026013871 in cell D236 of the “SSB price and tax” worksheet, matching the analytical record. Cell D41 reports 1.7498097519 for 2024. Appendix Table A3 documents the corresponding worksheet coordinates, reported values, and source concordance. The match establishes that the recorded observation came from the source, but it does not confirm a unit error, and the workbook offers no note explaining the anomaly. We therefore retain the reported values in the baseline and examine their influence through exclusion and winsorization, without applying an unverified conversion or replacement.

Entropy balancing provides a supplementary comparison alongside the continuous-exposure models [60]. In each outcome’s complete-case sample, we compare intensifiers with countries within the symmetric stability band. Countries whose excise shares fall by at least the cutoff are excluded. Control weights minimize relative entropy while matching the intensifiers’ first moments for the baseline outcome, 2021 log PPP GDP per capita, 2021 urbanization, and the 2022 excise share:

(4) $$min\Sigma_{i}w_{i}ln(\frac{w_{i}}{q_{i}}),\;\;s.t.\Sigma_{i}w_{i}c_{ik}={\bar{c}}_{k};\Sigma_{i}w_{i}=1;w_{i}\geq 0$$

In Equation (4), q_i_ represents uniform base weights, and c_ik_ represents the four balancing variables. We calculate the contrast as the unweighted mean change among intensifiers minus the weighted mean change among controls. This is a descriptive difference adjusted for observed covariates, not an average treatment effect. For each outcome and cutoff, we make 2000 stratified country-bootstrap attempts, resampling the groups separately and estimating new weights in every draw. A draw enters the interval calculation only when its maximum absolute imbalance is below 10^−5^ in control-standard-deviation units. Appendix A reports the number of retained draws and the effective control sample sizes. The resulting percentile intervals are conditional on successful balance and require particular caution when the intensifier group is small. The wild bootstrap uses seed 20,260,831; the script specifies the outcome-and-cutoff rule used to generate balancing seeds.

The processed data, executable code, model outputs, figure sources, source audit, and checksums used to reproduce the reported analyses are provided in the Supplementary Materials (File S1).

## 4. Results

### 4.1. Descriptive Patterns

Table 2 describes the global panel. For the 124 countries observed in both excise waves, the mean change was 0.349 percentage points and the median was zero. Thirteen countries recorded increases of at least two points, seven moved from no measured excise to a positive share, and eight recorded cuts of at least two points. The limited variation constrains precision. These data should not be treated as a global natural experiment in large statutory tax increases.

Between the two waves, the benchmark beverage’s mean PPP price increased, but its mean income burden changed little. Healthy-diet costs also rose in most countries amid substantial food inflation. Over the same period, the estimated prevalence of healthy-diet unaffordability and the three-year average prevalence of food insecurity both declined modestly. These aggregate patterns describe the setting for the adjusted models. They do not attribute the observed movements to SSB taxation.

### 4.2. Main First-Difference Estimates

The primary estimates in Table 3 associate a one-percentage-point increase in excise share with a 0.0023 increase in log SSB income burden. Uncertainty is substantial: the 95% HC3 confidence interval runs from −0.0349 to 0.0395, with HC3 *p* = 0.902 and wild-bootstrap *p* = 0.912. The approximate minimum detectable coefficient at 80% power is 0.0525 log points. For analytical objective 1, these results leave both the direction and the magnitude of the income-normalized association unresolved.

For PPP price, the untrimmed coefficient is 0.0799 (HC3 *p* = 0.190; wild *p* = 0.159), and its confidence interval crosses zero. The source-record checks in Section 4.3 materially affect this estimate. Even greater statistical precision would not make the coefficient a measure of tax pass-through, because retail price also enters the denominator of the excise-share exposure.

The food-system estimates are also too imprecise to establish the absence of adverse associations. The coefficient is −0.0026 for log healthy-diet cost, −0.4215 percentage points for unaffordability, and 0.1470 percentage points for the moderate/severe food-insecurity contrast between overlapping windows. Across the five outcomes, the smallest adjusted q-values are 0.317 for HC3 inference and 0.252 for the wild bootstrap. For the three food-system outcomes, the respective MDEs are 0.0048 log points, 0.6805 percentage points, and 0.3918 percentage points. Although no harmful association is estimated precisely, these results neither establish equivalence nor exclude changes that could matter for policy.

**Figure 1:**
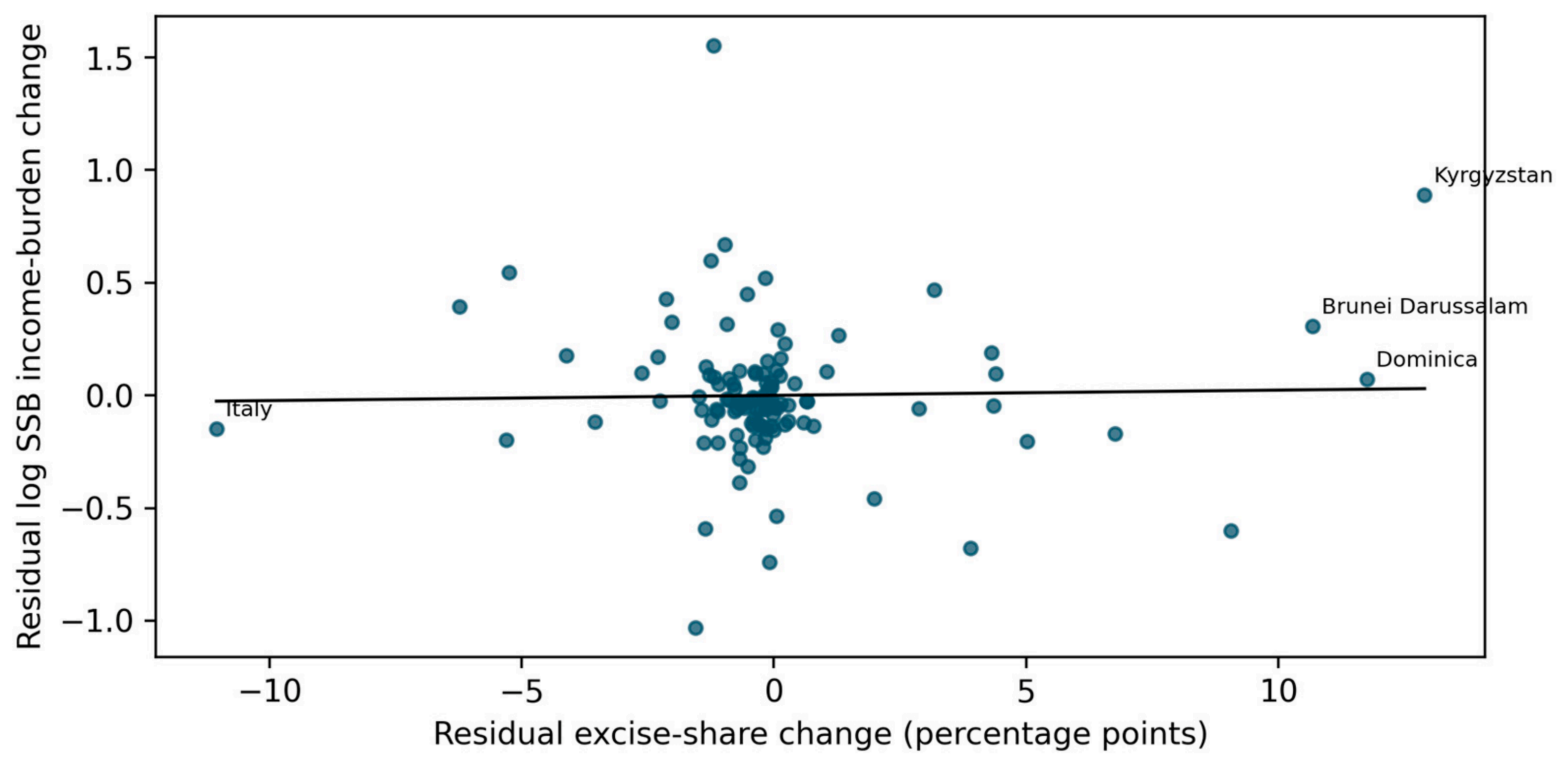
Partial-residual plot of excise-share change and SSB income-burden change, with a fitted line and selected country labels.

Figure 1 plots the residual changes in SSB income burden against those in excise share after removing their linear associations with the controls. The fitted line is nearly flat, with considerable dispersion across countries. This pattern accords with the wide confidence interval in Table 3.

**Figure 2:**
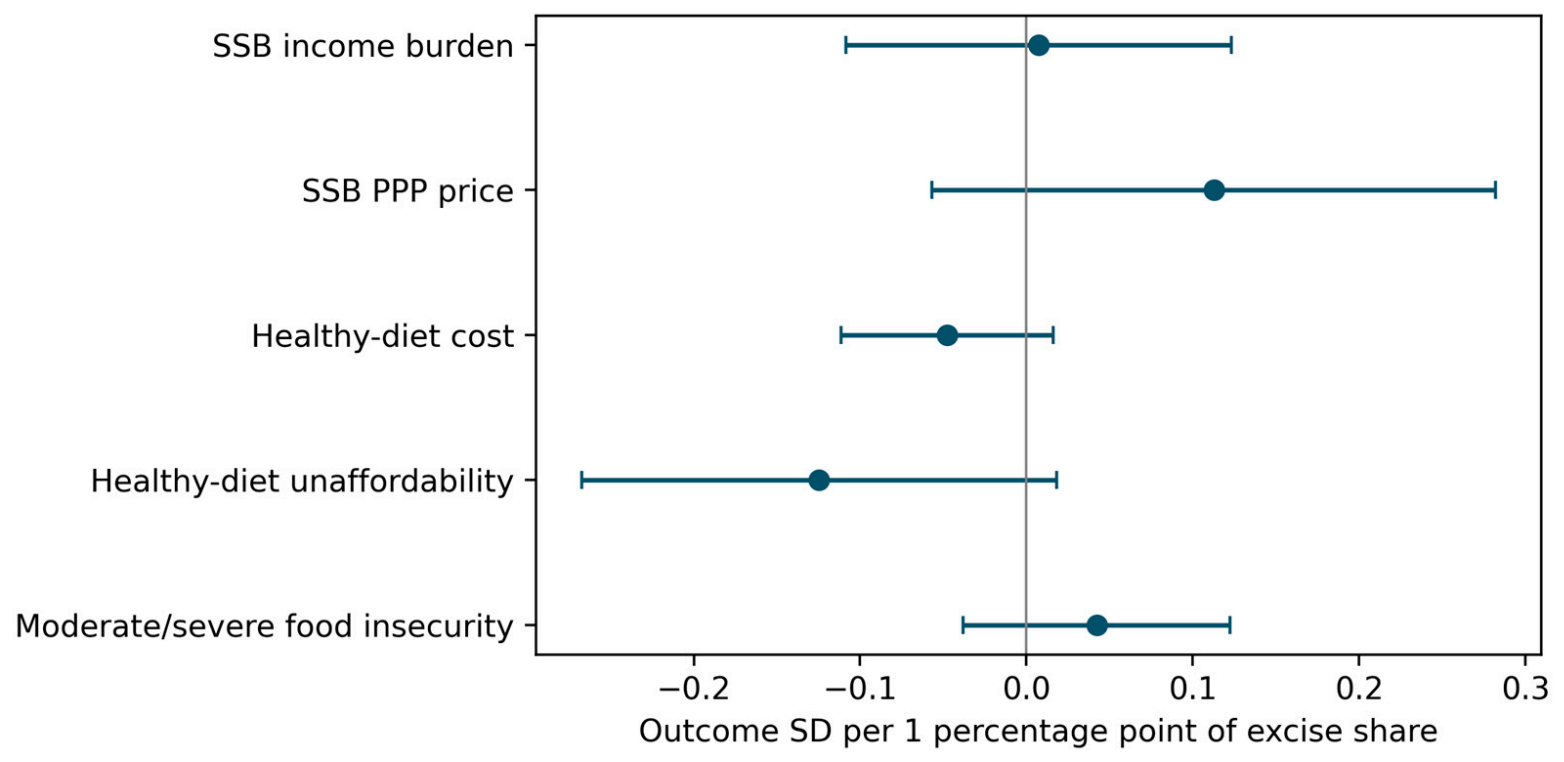
Standardized coefficients and 95% HC3 confidence intervals for targeted affordability and food-system safeguard outcomes.

Figure 2 expresses each coefficient and its 95% HC3 confidence limits relative to the standard deviation of the outcome in its estimation sample. The transformation allows comparisons across outcomes while preserving statistical uncertainty. All intervals cross zero. Standardizing the estimates does not give these descriptive associations a causal interpretation or turn them into an equivalence test.

### 4.3. Principal Sensitivity Checks

Table 4 presents the checks most relevant to the main interpretation: removing the contemporaneous food-inflation control and excluding the anomalous PPP-price observation. Without the food-CPI control, the income-burden coefficient remains imprecise (β = 0.0031; *p* = 0.860). Appendix Table A1 reports every sensitivity specification for all five outcomes, and Table A1 also summarizes the leave-one-country-out analysis.

Excluding Sierra Leone reduces the PPP-price coefficient from 0.0799 to 0.0238 (*p* = 0.263). Winsorizing within the complete-case sample yields 0.0191 (*p* = 0.253; Appendix Table A1). Direct source inspection confirms transcription of the unusual 2022 value but does not validate the value itself. The variation across these specifications therefore matters more for interpretation than the untrimmed coefficient alone.

After winsorization within the complete-case samples, the coefficients are −0.0024 for log healthy-diet cost (*p* = 0.211), −0.2454 percentage points for healthy-diet unaffordability (*p* = 0.152), and 0.2067 percentage points for food insecurity (*p* = 0.280). Appendix Table A1 includes all three estimates. The remaining specifications are also imprecise. Agreement in this respect does not exclude adverse changes.

### 4.4. Entropy-Balanced Intensifier Comparison

For the two-point entropy-balanced comparison in Table 5, effective control sample sizes range from 60.2 to 71.3, and the largest control weight is 0.032. Depending on the outcome, only 8–13 intensifiers are available. Balancing the observed first moments cannot remove selection on unmeasured characteristics or establish causality.

The adjusted difference in log SSB income burden is 0.0356, with a 95% bootstrap interval of −0.1585 to 0.2367. All five outcome intervals include zero at the two-point cutoff. Appendix Table A2 repeats the comparisons with cutoffs from 1 to 5 percentage points to assess how the descriptive grouping affects the results. The continuous-exposure models in Table 3 remain the primary analysis. At the five-point cutoff, the positive untrimmed PPP-price contrast depends on the inclusion of Sierra Leone (Appendix A.2). Several bootstrap comparisons also lose substantial numbers of draws because balance cannot be achieved.

### 4.5. Supplementary Associations with Tax Design in 2024

To address analytical objective 3, Table 6 relates log SSB income burden in 2024 separately to the contemporaneous excise share and the design checklist. Both cross-sectional models adjust for baseline income, urbanization, and region. They describe whether the documented tax structure is associated with affordability at the endpoint, rather than explaining the 2022–2024 changes or evaluating individual reforms. The full-sample coefficients are positive but imprecise. Among countries with an excise, the checklist coefficient becomes negative and remains imprecise (β = −0.0563; *p* = 0.316). Together with the component estimates in Appendix Table A4, this sign change indicates that the equally weighted checklist should not be treated as a validated measure of policy effectiveness.

## 5. Discussion

Our estimates leave the association between excise-share changes and SSB income burden uncertain. The PPP-price result also depends on an anomalous source observation. This evidence addresses a different question from evaluations of discrete tax reforms. For example, the multi-city US study examines implemented taxes and purchasing responses [64], while the Irish hospitality study focuses on a particular retail setting [65]. We pool comparatively small changes in a tax share whose denominator is retail price across countries. Differences in the exposure, outcomes, and retail coverage prevent a direct numerical comparison with those evaluations.

### 5.1. What the Two-Year Global Evidence Does—And Does Not—Show

The width of the confidence intervals is central to interpreting the findings. Average excise-share movements are small, and the available sample differs by outcome, leaving limited precision for modest associations. Reviews of economic evaluations discuss related difficulties in attributing effects and assessing equity [66]. Evidence from Europe and Saudi Arabia also shows why statutory design and consumption patterns deserve attention [67,68]. A stable income-burden indicator or an uncertain price coefficient cannot, by itself, establish that a specific reform was unsuccessful.

The measurement choices further distinguish this analysis from evaluations of individual policies. Because income burden incorporates average income growth, it can remain stable as beverage prices rise. Changes in excise share likewise reflect movements in both the tax amount and retail price. Inflation may reduce the real value of a nominal specific levy even when the statutory rate is unchanged. PPP conversion affects the price outcome, but a common currency conversion cancels from a consistently measured tax-to-price ratio. Manufacturers may also respond through reformulation. The UK findings show that sugar content can change appreciably despite only partial price pass-through [22].

The difference between the untrimmed price estimate and the estimate excluding Sierra Leone demonstrates how a single source observation can affect the results. Direct inspection found the unusual PPP value in the WHO workbook, ruling out a mismatch introduced during extraction. The reason for that value remains unknown. Reporting both the recorded observation and an exclusion analysis exposes its influence while avoiding an unsupported replacement.

### 5.2. Food Security, Regressivity, and the Missing Revenue Side

The broader food-system outcomes provide insufficient evidence either of harmful spillovers or of safety. Healthy-diet cost and unaffordability describe basket prices and economic access, whereas food insecurity captures experienced difficulty obtaining food over less closely aligned observation periods. International healthy-diet comparisons also depend on the price inputs and affordability standards used [69,70,71]. The contrast between overlapping food-insecurity windows is therefore best used to identify contexts requiring closer investigation, rather than to measure a short-run response to taxation.

SSBs offer a useful case because governments widely target them with taxes and comparable tax, price, and affordability indicators are available. This choice does not imply that beverages dominate household food budgets. Staples and other regularly purchased foods may account for much more spending. Moreover, the benchmark burden divides the cost of a hypothetical fixed purchase by GDP per capita. Actual expenditure varies with quantities, brands, substitution, and household income. The indicator therefore cannot reveal whether the tax is regressive or whether beverage purchases displace spending on healthy foods [13,17,19,41].

How governments spend tax receipts could affect food access, but the data do not observe this channel. Investment in water, school nutrition, or targeted assistance may change households’ net benefits. Our checklist records only whether revenue is earmarked, without identifying the amount or the beneficiaries. Recent evaluations examine health outcomes outside the present study’s scope [72,73,74], and international assessments and methodological reviews identify difficulties in attributing outcomes across settings [75,76]. Evidence on affordability in India [77], alongside cross-regional and simulation studies [78,79,80], further supports measuring tax design, consumption, and revenue use separately. The present estimates cannot resolve their effects on household welfare or long-term health.

### 5.3. Design Implications for Ministries of Finance and Health

The empirical findings identify a need for closer monitoring. Recommendations about tax design rely on the wider literature, since these models do not estimate causal policy effects. Monitoring should record statutory rates and tax amounts alongside benchmark affordability [37,38,39,40]. Indexation can protect the real value of a specific levy against inflation, but maintaining its strength relative to income also requires attention to income growth [2,37,38]. Where benchmark burden remains flat, separating price movements from income growth would clarify whether beverage prices have nevertheless increased.

Specific and sugar-based taxes can tie liability more closely to the health concern being addressed than ad valorem taxes [1,5,16,17]. With a tiered structure, manufacturers may reformulate products before the levy reaches consumers, so price pass-through can capture only part of the response [22]. Evaluation should accordingly measure sugar content, package size, sales, and reformulation alongside prices and affordability.

Keeping unsweetened water outside the SSB excise preserves its position as an untaxed, healthier alternative. Extending the tax to water can weaken the incentive to substitute away from sugary drinks and undermine the food-security rationale. Policy databases document water taxation across countries, making the treatment of substitutes a practical design issue [2,5].

Decisions about revenue use should accompany decisions about the tax itself. Funding water infrastructure or support for healthy foods could connect a beverage tax to broader food-system objectives. Simulations combining unhealthy-food taxes with subsidies for minimally processed foods illustrate why these joint measures require direct evaluation [41,42].

Monitoring should distinguish the statutory tax structure and amounts collected from product prices, affordability relative to income, and observed dietary or food-access outcomes. Household expenditure data and information on revenue use are needed to connect these measures. The global indicators help identify settings for further investigation; the short panel, source anomaly, and uncertainty documented here constrain what can be concluded from them.

## 6. Limitations and Future Research

The two-wave design cannot establish pre-policy trends, and limited variation in exposure constrains precision. Excise share is a tax-to-price ratio, not an exogenous measure of a statutory reform. Other nutrition policies, exchange-rate changes, drought, conflict, and transfers may vary over time and confound the estimated associations. Food inflation may itself respond to a wider policy package. Removing the contemporaneous food-CPI control changes what the model adjusts for but does not resolve these identification problems.

Complete-case samples vary by outcome and restrict geographic representativeness; Appendix Table A5 compares included and excluded countries. Neither the benchmark beverage nor GDP per capita represents the household consumption basket or the distribution of disposable income. The Sierra Leone PPP-price anomaly also remains unexplained despite confirmation that the panel matches the source. Aggregate country data cannot distinguish substitution, gross tax payments, net fiscal benefits, or responses across income groups.

The overlapping three-year food-insecurity windows prevent identification of an annual response or one timed to a policy change. Similarly, the design checklist assigns equal weights to legal features without measuring how intensively they are implemented. Entropy balancing aligns observed first moments only. With few intensifiers, the bootstrap intervals depend heavily on overlap and the feasibility of obtaining balance. These limitations rule out causal interpretations, distributional conclusions, and claims of equivalence.

Additional WHO waves would allow a longer panel with country and year fixed effects, policy-specific treatment dates, and event-time analysis. Linking tax design to household purchase and income data would be especially valuable, as it could distinguish gross tax payments from substitution, health gains, and benefits financed by tax revenue. Further work should compare sugar-based tiers and automatic adjustment with simple volumetric and ad valorem designs during high inflation. Such evaluations should also incorporate local water access: an exemption for bottled water is useful only where households can obtain safe, affordable substitutes.

## 7. Conclusions

In the 2022–2024 country samples, the associations of excise-share changes with SSB income burden and the three food-system outcomes remain imprecise after adjustment for multiple testing. The PPP-price estimate is sensitive to an anomalous source observation, and the price outcome is mechanically related to the denominator of the exposure. These findings establish neither that SSB taxes are ineffective nor that food-security risks are absent.

Monitoring beverage affordability together with healthy-diet access and experienced food insecurity can identify priorities for closer evaluation. Assessing effects on household food budgets and welfare will require longer panels, reform-specific dates, household purchase and income data, and records of how tax revenue is used.

## Figures and Tables

**Table 1:** Open data sources and analytical roles. Exact URLs and retrieval dates are reproduced in the data workbook.

Source/Release	Coverage Used	Variables	Analytical Role
WHO GHO [4] (July 2024 release)	195-country frame; 2022 and 2024	SSB price, tax decomposition, affordability, legal design	Exposure and targeted outcomes
FAO CoAHD [51] (July 2026 release)	Country-year, 2022 and 2024	Healthy-diet cost; population unable to afford	Food-system safeguards
FAOSTAT Food Security (July 2026 release)	Published 3-year averages	Moderate/severe food insecurity	Broad access safeguard
FAOSTAT CPI (July 2026 release)	Monthly data annualized to 2022/2024	Food Consumer Price Index	General food inflation control
World Development Indicators [52]	2021 baseline and regional metadata	PPP GDP per capita, urbanization, population, region	Structural controls and crosswalk

Note: The source register records release dates, original retrieval dates, and country-linkage information. The study uses outcome-specific complete-case samples within the 195-country frame.

**Table 2:** Descriptive statistics. Counts reflect variable availability before regression-specific controls.

Variable	n	Mean	SD	Median	Min	Max
2024 excise share (%)	160	5.564	7.978	2.398	0.000	49.500
Δ excise share (pp)	124	0.349	2.865	0.000	−11.037	13.651
Δ total-tax share (pp)	124	0.211	3.791	0.000	−13.006	13.651
Δ log SSB PPP price	121	0.165	0.690	0.101	−1.956	6.511
Δ log SSB income burden	120	0.027	0.315	−0.007	−1.091	1.896
Δ log healthy-diet cost	156	0.113	0.058	0.112	−0.196	0.309
Δ healthy-diet unaffordability (pp)	144	−1.352	3.428	−0.600	−16.200	14.700
Δ moderate/severe food insecurity (pp)	130	−0.455	3.629	−0.100	−11.500	12.600
Health-aligned design score (0–6)	195	1.790	1.240	1.000	0.000	5.000

Note: n denotes available observations before regression-specific exclusions; SD denotes standard deviation; pp denotes percentage points. Changes compare 2022 and 2024, except food insecurity, which contrasts overlapping three-year windows.

**Table 3:** Main first-difference estimates, 2022–2024. Models include baseline income, urbanization, food-CPI change, and region indicators. Wild *p* uses 9999 draws; BH q spans all five HC3 tests.

Outcome	n	β	HC3 SE	HC3 *p*	Wild *p*	BH q	MDE
SSB burden	112	0.0023	0.0188	0.902	0.912	0.902	0.0525
SSB PPP price	113	0.0799	0.0606	0.190	0.159	0.317	0.1696
Healthy-diet cost	102	−0.0026	0.0017	0.140	0.149	0.317	0.0048
Diet unaffordability	95	−0.4215	0.2430	0.086	0.092	0.317	0.6805
Food insecurity	83	0.1470	0.1399	0.297	0.202	0.371	0.3918

Note: β is per one-percentage-point increase in excise share. The first three outcomes are log changes; the final two are prevalence changes in percentage points. MDE is the approximate 80% minimum detectable coefficient. BH q adjusts the five HC3 tests.

**Table 4:** Principal sensitivity checks. Complete results appear in Appendix Table A1.

Outcome	Specification	n	β	HC3 SE	*p*
SSB burden	No food-CPI control	115	0.0031	0.0173	0.860
SSB PPP price	No food-CPI control	116	0.0814	0.0628	0.198
SSB PPP price	Exclude Sierra Leone	112	0.0238	0.0211	0.263
Healthy-diet cost	No food-CPI control	103	−0.0023	0.0020	0.237
Diet unaffordability	No food-CPI control	97	−0.3994	0.2710	0.144
Food insecurity	No food-CPI control	84	0.1803	0.1133	0.116

Note: β is an adjusted association per one-percentage-point increase in excise share. HC3 *p*-values are unadjusted sensitivity diagnostics.

**Table 5:** Entropy-balanced adjusted comparisons of intensifiers (≥2 pp) and stable countries (|change| < 2 pp).

Outcome	n (I/S)	Adjusted Difference	95% Bootstrap CI	Control ESS	Max Weight
SSB burden	107 (13/94)	0.0356	[−0.1585, 0.2367]	71.3	0.025
SSB PPP price	108 (13/95)	0.5541	[−0.0541, 1.5847]	66.7	0.030
Healthy-diet cost	96 (12/84)	−0.0092	[−0.0381, 0.0203]	66.9	0.025
Diet unaffordability	90 (10/80)	−2.3278	[−5.5701, 0.3798]	60.2	0.032
Food insecurity	79 (8/71)	0.8404	[−1.3185, 3.0166]	63.4	0.030

Note: I = intensifier; S = stable country; ESS = effective sample size. Percentile intervals use 2000 stratified country-bootstrap attempts with weights re-estimated in each draw. See Appendix Table A2 for successful draws and overlap diagnostics.

**Table 6:** Descriptive 2024 models of log SSB income burden and design-checklist sensitivity.

Exposure	Sample	n	β	HC3 SE	*p*
excise share 2024	Full sample	149	0.0160	0.0093	0.088
health design score	Full sample	149	0.0413	0.0259	0.114
health design score	Excise countries only	96	−0.0563	0.0558	0.316

Note: Each row is a separate model of log SSB income burden, controlling for baseline income, urbanization, and region. The checklist coefficient is per additional documented design feature; its *p*-values are exploratory.

## Data Availability

The accompanying replication package provides the processed country panel, variable dictionary, country crosswalk, WHO-derived records, source URLs and recorded retrieval dates, executable analysis code, and model and figure outputs. It enables reproduction of the reported analysis from the frozen panel. The original FAO and World Bank bulk inputs and their extraction pipeline are not included; the workbook records the institutional source links. The source audit documents the direct WHO workbook check for Sierra Leone. The study uses no household or individual records.

## Supplementary Materials

The following supporting information can be downloaded at: https://www.mdpi.com/article/10.3390/foods15183343/s1, File S1: Foods_SSB_Replication_Package.zip (processed country panel, variable dictionary, country crosswalk, WHO-derived records, source register, executable analysis and figure code, model outputs, figures, source-audit notes, environment record, and SHA-256 checksums).

## Appendix A. Supplementary Analyses

### Appendix A.1. Specification and Influence Checks

Table A1 presents the five sensitivity specifications for each outcome. Coefficients correspond to a one-percentage-point increase in the tax share specified in the row. Log and prevalence outcomes retain their original units. The sensitivity *p*-values are unadjusted and exploratory.

**Table A1:** Sensitivity specifications across all outcomes.

Outcome	Specification	n	β	HC3 SE	*p*
SSB income burden	No food-CPI control	115	0.0031	0.0173	0.860
SSB income burden	Exclude Sierra Leone	111	0.0092	0.0177	0.606
SSB income burden	Exclude excise cuts	104	0.0089	0.0238	0.711
SSB income burden	2.5/97.5 winsorized	112	−0.0046	0.0170	0.787
SSB income burden	Total-tax change	112	0.0043	0.0118	0.718
SSB PPP price	No food-CPI control	116	0.0814	0.0628	0.198
SSB PPP price	Exclude Sierra Leone	112	0.0238	0.0211	0.263
SSB PPP price	Exclude excise cuts	105	0.0977	0.0812	0.232
SSB PPP price	2.5/97.5 winsorized	113	0.0191	0.0166	0.253
SSB PPP price	Total-tax change	113	0.0368	0.0264	0.165
Healthy-diet cost	No food-CPI control	103	−0.0023	0.0020	0.237
Healthy-diet cost	Exclude Sierra Leone	101	−0.0028	0.0018	0.124
Healthy-diet cost	Exclude excise cuts	95	−0.0025	0.0025	0.315
Healthy-diet cost	2.5/97.5 winsorized	102	−0.0024	0.0019	0.211
Healthy-diet cost	Total-tax change	102	−0.0023	0.0013	0.095
Healthy-diet unaffordability	No food-CPI control	97	−0.3994	0.2710	0.144
Healthy-diet unaffordability	Exclude Sierra Leone	94	−0.4564	0.2677	0.092
Healthy-diet unaffordability	Exclude excise cuts	88	−0.6140	0.3747	0.105
Healthy-diet unaffordability	2.5/97.5 winsorized	95	−0.2454	0.1699	0.152
Healthy-diet unaffordability	Total-tax change	95	−0.2710	0.1524	0.079
Moderate/severe food insecurity	No food-CPI control	84	0.1803	0.1133	0.116
Moderate/severe food insecurity	Exclude Sierra Leone	82	0.1644	0.1403	0.245
Moderate/severe food insecurity	Exclude excise cuts	78	0.1492	0.2356	0.529
Moderate/severe food insecurity	2.5/97.5 winsorized	83	0.2067	0.1897	0.280
Moderate/severe food insecurity	Total-tax change	83	0.0923	0.0729	0.209

Across the 112 leave-one-country-out income-burden models, coefficients range from −0.0131 to 0.0092, and the smallest HC3 *p*-value is 0.335. The file outputs/leave_one_out.csv contains the country-level results. Winsorization uses quantiles from each complete-case sample, so its results need not match trimming based on the full 195-country frame.

### Appendix A.2. Sensitivity to the Intensification Cutoff

Table A2 compares intensifiers, defined as Δ excise share ≥ c, with stable countries, defined as |Δ excise share| < c, at cutoffs of 1, 2, 3, and 5 percentage points. Countries whose excise shares fall by at least c are excluded. Each specification balances the same four types of covariates. Interval calculations include only successful draws, and the reported counts identify attempts in which balance could not be achieved.

**Table A2:** Entropy-balanced comparisons across intensification cutoffs and outcomes.

Outcome	Cutoff	I/S	Difference	95% Bootstrap CI	Control ESS	Valid Draws
SSB income burden	1	14/87	0.0575	[−0.1309, 0.2633]	59.4	1996/2000
SSB income burden	2	13/94	0.0356	[−0.1585, 0.2367]	71.3	2000/2000
SSB income burden	3	13/98	0.0304	[−0.1695, 0.2252]	72.1	2000/2000
SSB income burden	5	6/105	0.1037	[−0.1855, 0.4325]	72.5	1945/2000
SSB PPP price	1	14/88	0.5406	[−0.0482, 1.4691]	57.5	1997/2000
SSB PPP price	2	13/95	0.5541	[−0.0541, 1.5847]	66.7	1996/2000
SSB PPP price	3	13/99	0.5532	[−0.0784, 1.6081]	67.7	1997/2000
SSB PPP price	5	6/106	1.2128	[0.0338, 2.9184]	54.1	1887/2000
Healthy-diet cost	1	13/77	−0.0121	[−0.0424, 0.0211]	56.8	1995/2000
Healthy-diet cost	2	12/84	−0.0092	[−0.0381, 0.0203]	66.9	1999/2000
Healthy-diet cost	3	12/87	−0.0095	[−0.0401, 0.0191]	68.9	1998/2000
Healthy-diet cost	5	5/94	−0.0261	[−0.0715, 0.0210]	60.5	1963/2000
Healthy-diet unaffordability	1	11/73	−2.1653	[−5.3541, 0.5811]	48.3	1990/2000
Healthy-diet unaffordability	2	10/80	−2.3278	[−5.5701, 0.3798]	60.2	1996/2000
Healthy-diet unaffordability	3	10/83	−2.3109	[−5.6845, 0.4240]	63.0	1997/2000
Healthy-diet unaffordability	5	3/90	−3.9163	[−9.4044, 1.3879]	24.4	1461/2000
Moderate/severe food insecurity	1	8/66	0.7457	[−1.4577, 2.8252]	59.2	1995/2000
Moderate/severe food insecurity	2	8/71	0.8404	[−1.3185, 3.0166]	63.4	1995/2000
Moderate/severe food insecurity	3	8/73	0.8367	[−1.5074, 2.8779]	65.1	1988/2000
Moderate/severe food insecurity	5	2/79	−0.9494	[−4.8101, 1.5046]	17.8	1394/2000

At some cutoffs, exploratory intervals exclude zero. However, changing the cutoff also changes group membership, and leaves small intensifier samples across multiple unadjusted comparisons. Table A2 presents all cutoffs so that this variation can be assessed without selecting a favorable specification. These comparisons supplement the primary continuous-exposure models.

At the five-point cutoff, the untrimmed PPP-price contrast is 1.2128 (95% interval 0.0338 to 2.9184), based on 6 intensifiers. After excluding Sierra Leone, it falls to 0.2362 (95% interval −0.0282 to 0.5497), with 5 intensifiers and 106 stable countries. Balance is achieved in 1996 of 2000 bootstrap attempts. The positive untrimmed result is thus sensitive to the anomalous observation and provides no basis for a general conclusion about prices or pass-through.

The lowest proportion of retained bootstrap draws is 69.7%. The accompanying output CSV reports maximum standardized imbalance and maximum weights as well. Because the intervals are conditional on achieving balance, comparisons at the highest cutoffs require particular caution: only a few countries meet the intensifier definition.

### Appendix A.3. Sierra Leone Source Concordance

A direct inspection of the WHO workbook located the cells listed below in the “SSB price and tax” worksheet. Their agreement with the analytical panel confirms source concordance. It does not validate the underlying PPP conversion or explain the anomalous observation.

**Table A3:** WHO source concordance for Sierra Leone benchmark SSB prices, 2022 and 2024.

Year	Source Row	Reported Price/Currency	PPP Price	USD Price
2022	236	10/SLE	0.0026013871	0.7204610951
2024	41	10/SLE	1.7498097519	0.4444108667

The WHO source provides no explanatory note for the 2022 PPP value. We retain the recorded observation and report the effect of excluding the country, without introducing an assumed conversion factor. The replication package supplies the audit URL, worksheet coordinates, retrieval date, and source checksum.

### Appendix A.4. Individual Tax-Design Components

**Table A4:** Associations with 2024 log SSB income burden among countries with an excise.

Feature	n	β	HC3 SE	*p*
sugar tier	96	−0.0799	0.1027	0.439
automatic adjustment	96	−0.2219	0.1479	0.137
water exempt	96	−0.1177	0.0975	0.231
specific excise	96	0.0239	0.1256	0.849
earmarked	96	−0.0655	0.1177	0.579
health design score	96	−0.0563	0.0558	0.316

Rows report separate models adjusted for baseline log income, urbanization, and region. Individual components are binary, while the checklist runs from 0 to 6. These exploratory *p*-values do not establish the effectiveness of any component.

### Appendix A.5. Sample Composition

**Table A5:** Baseline characteristics of included and excluded countries.

Outcome	Group	n	Median GDP PPP	Median Urban %
SSB burden	Included	112	16,061	61.1
SSB burden	Excluded	83	13,459	64.2
SSB PPP price	Included	113	15,809	61.0
SSB PPP price	Excluded	82	13,978	64.7
Healthy-diet cost	Included	102	16,315	62.5
Healthy-diet cost	Excluded	93	13,459	62.8
Diet unaffordability	Included	95	15,401	61.1
Diet unaffordability	Excluded	100	15,746	63.7
Food insecurity	Included	83	18,076	59.4
Food insecurity	Excluded	112	13,978	64.7

GDP refers to 2021 PPP GDP per capita in the archived panel. Group medians use available observations, without imputing missing values. The excluded group consists of countries in the 195-country frame that do not enter the complete-case regression sample for the relevant outcome.
